# Supplementary figures and images for: Health system barriers to hypertension care in Peru: Rapid assessment to inform organizational-level change
Source: PLOS Glob Public Health. 2024 Aug 19;4(8):e0002404. doi: 10.1371/journal.pgph.0002404 (PMC11332938; doi:10.1371/journal.pgph.0002404)

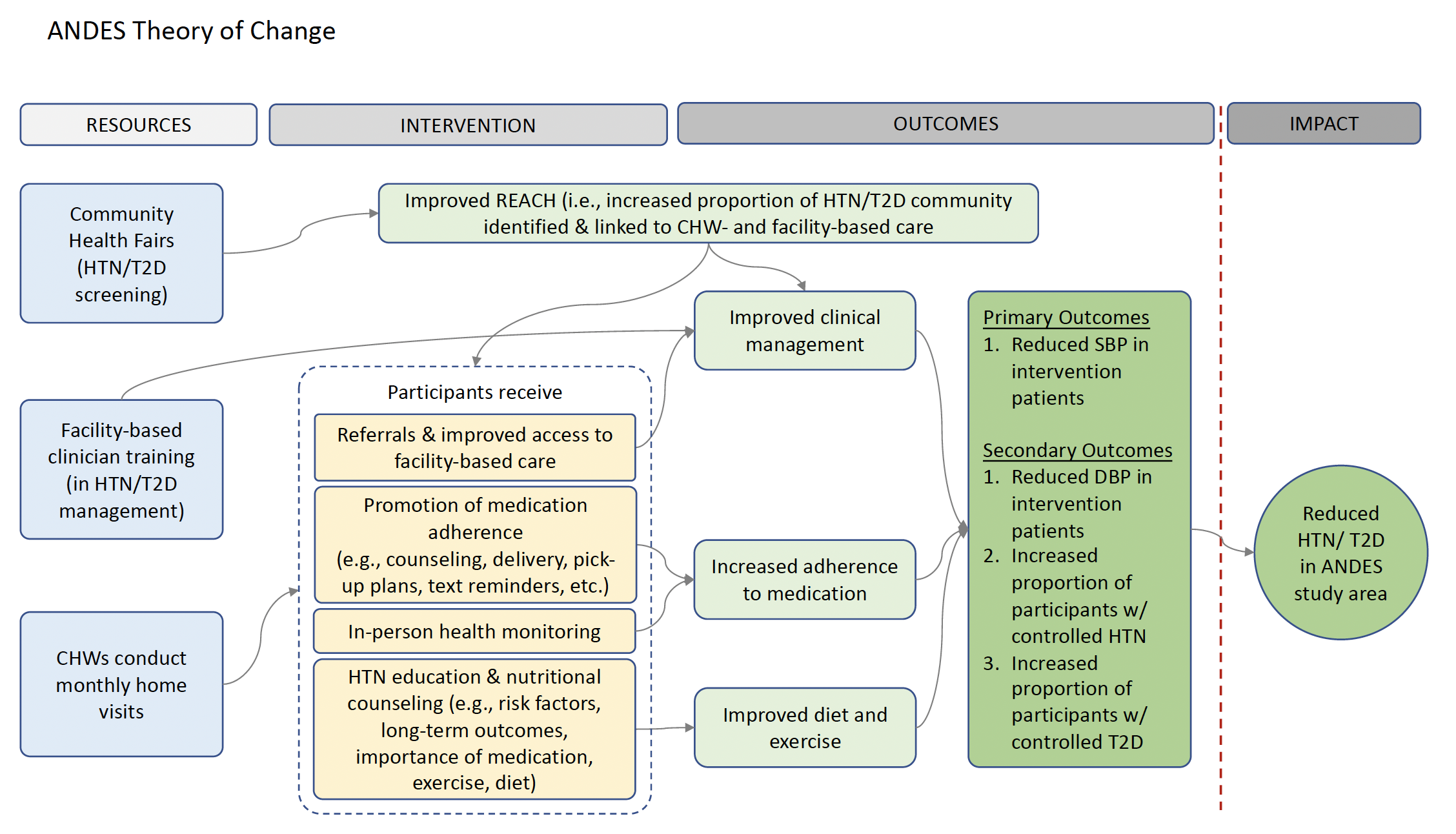

Supplement: S1 Fig — (TIFF) [file pgph.0002404.s001.tiff]

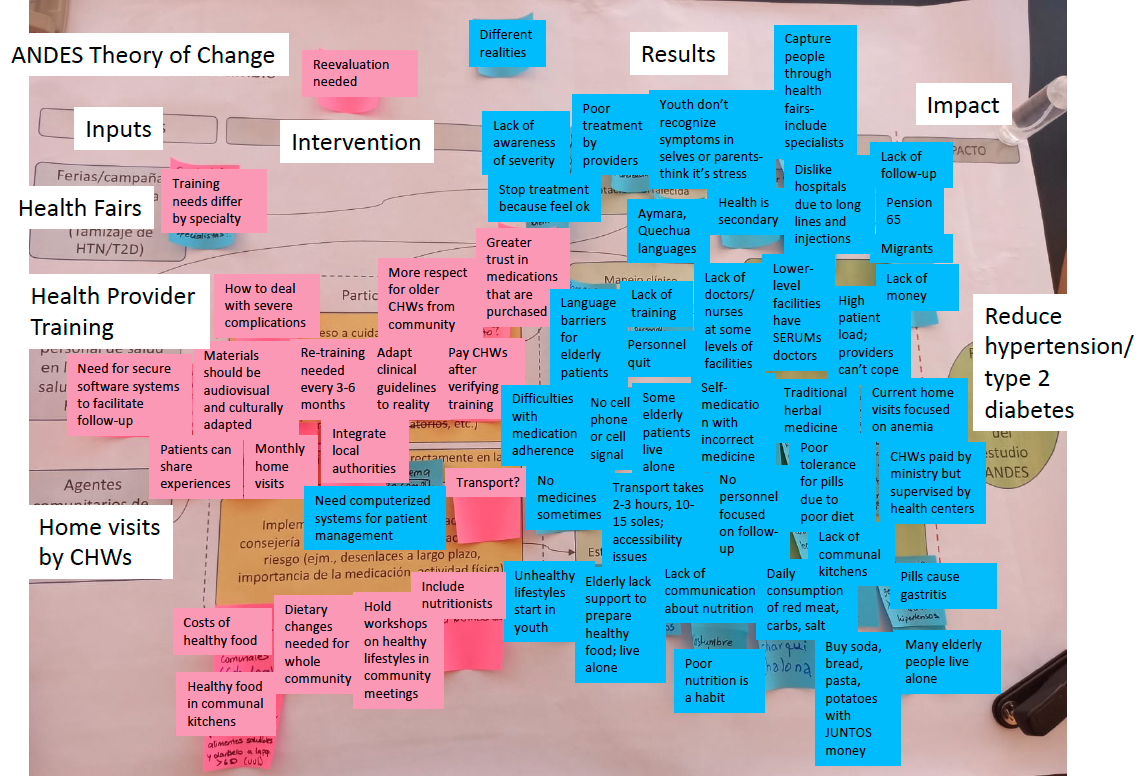

Supplement: S2 Fig — (TIFF) [file pgph.0002404.s002.tiff]
