# Supplementary material for: Health system barriers to hypertension care in Peru: Rapid assessment to inform organizational-level change
Source: PLOS Glob Public Health. 2024 Aug 19;4(8):e0002404. doi: 10.1371/journal.pgph.0002404 (PMC11332938; doi:10.1371/journal.pgph.0002404)
Supplement: S1 Table — (PDF) [file pgph.0002404.s003.pdf]

S1 Table. Characteristics of workshop participants.

	Total	# Rural	# Urban	# hospitals	# health clinics	# MINSA or DIRESA
Nurses	15	6	9	3	11	1
Doctors	10	3	7	1	5	4
Dentists	6	3	3	0	6	0
Nutritionists	1	0	1	0	1	0
Biologists	1	1	0	0	1	0
Midwives	1	0	1	0	0	1
Total	34	13	21	4	24	6

* Provinces covered included Carabaya, Yunguyo, Sandia, Collao, Melgar, Azángaro, Lampa, Chucuito, Puno, San Roman, Huancané
