# Supplementary material for: Health system barriers to hypertension care in Peru: Rapid assessment to inform organizational-level change
Source: PLOS Glob Public Health. 2024 Aug 19;4(8):e0002404. doi: 10.1371/journal.pgph.0002404 (PMC11332938; doi:10.1371/journal.pgph.0002404)
Supplement: S2 Table — (PDF) [file pgph.0002404.s004.pdf]

S2 Table. Characteristics of in-depth interview participants.

	**Total**	**# Rural**	**# Urban**	**1 year or less at facility**	**>1 year at facility**	**Leadership position**	**Non-leadership**
Nurses	7	6	1	2	5	1	6
Doctors	12	9	3	9	3	2	10
Midwives	1	1	0	0	1	1	0
Dentists	1	1	0	0	1	1	0
Total	21	17	4	11	10	5	16
