# Supplementary material for: Health system barriers to hypertension care in Peru: Rapid assessment to inform organizational-level change
Source: PLOS Glob Public Health. 2024 Aug 19;4(8):e0002404. doi: 10.1371/journal.pgph.0002404 (PMC11332938; doi:10.1371/journal.pgph.0002404)
Supplement: S1 File — (PDF) [file pgph.0002404.s005.pdf]

<u>ANDES Interview Guide – Health Professionals</u>

Goals:

Understand how the providers interact with community health workers and community members. Also, explore previous experiences with health fairs and treatment of people with HTN/DM.

Questions:

1. Ice Breaker: Tell me about your typical work day; for example, tell me about the people you care for at the health center.
   - *You can explore the most common diseases, grouped by age, sex, distance of health center from communities, etc.*
   - *Depending on the first interaction, another question or statement may be used to break the ice.*

2. What is your role in the health center?
   - *Do not limit yourself to the profession, but explore the activities they do*
   - *What activity do you do the most? What activity takes you the most time? What activities are the most complicated?*

3. What training have you received to care for patients who come to this center with hypertension or diabetes?
   - *Explore with questions such as: What training have you received to measure blood pressure or blood glucose? How is screening for hypertension and diabetes performed? What treatment should people with hypertension and diabetes receive?*

KNOWLEDGE AND PRACTICES ABOUT HYPERTENSION AND DIABETES

4. At your health center, after someone is diagnosed with hypertension or diabetes, what type of follow-up is done with patients?
   - ➔ **Explore:** Who's tracking? How are follow-ups scheduled? How do you coordinate with the patient? How do you ensure that the patient returns for those follow-ups?

5. What are the regulatory documents of the (MINSA/DIRESA or the health center) that guide the care of people with hypertension and/or diabetes?
   - *Explore if they know whether there are any clinical practice guidelines or standardized procedures.*

   ➔ If they DON'T know, **ask** : Do you think there should be any documents?
   - *Explore: What? How? Why?*

6. At your health center or for DIRESA Puno, what are the goals for controlling hypertension and/or diabetes?
   - *Explore: Who and how are goals set?*
   - *Ask: How is progress toward the goal for this month/year?*

   ➔ If they DON'T know, **ask** : Do you think a goal should be established?
   - *Explore: What? How? Why?*

7. What recommendations do you give to patients with hypertension and/or diabetes for self-care?
   - *Explore: drug treatments*
   - *Explore: recommendations on diet, physical activity or weight reduction*
   - *Explore: home care recommendations*

8. What are the challenges of treating patients with hypertension or diabetes?
   - ➔ **Explore aspects of the system:** e.g. availability of equipment, medication, costs
   - ➔ **Explore aspects of the person:** e.g. Patient acceptance, medication compliance, etc.

9. What makes it easier for you or the health center to treat patients with hypertension or diabetes?
   ➔ **Explore aspects of the system:** e.g. Quick detection, timely medications, etc.
   ➔ **Explore aspects of the person:** e.g. Family support, medication adherence

10. Could you tell me the story of someone with well-controlled hypertension or diabetes?
    – *If they do not remember a specific story, ask to describe characteristics of patients with well-controlled hypertension or diabetes.*
    – *Ask about the frequency of patients with controlled hypertension or diabetes in the health center/community*

11. Could you tell me the story of someone with uncontrolled hypertension?
    – *If they do not remember a specific story, ask to describe characteristics of patients with uncontrolled hypertension or diabetes.*
    – *Ask about the frequency of patients with uncontrolled hypertension or diabetes in the health center/community*

12. What are the differences between people with controlled hypertension and/or diabetes and those who are not?
    ➔ **Explore** personal factors, i.e. family support, lifestyle, peer support, motivations to follow treatment plan

RELATIONSHIP WITH COMMUNITY HEALTH AGENTS

13. How do you interact with community health agents?
    ➔ If **THEY DO NOT** INTERACT DIRECTLY, **ask :** What area or person is in charge of coordinating with community agents?

14. How do you think community health agents interact with patients with hypertension or diabetes? What type of treatment or follow-up do they provide?
    ➔ If THERE IS **NO** INTERACTION OR FOLLOW-UP, **ask:** Assuming that community agents can carry out some follow-up on patients with hypertension or diabetes, what activities do you think they could carry out?
       – *Explore activities that can be done with the patient, with the family or in the community*

15. What do you think would help community agents be able to provide care to patients with hypertension or diabetes?
       – *Explore training, materials, skills, etc.*

16. What do you think would make it difficult for community health workers to be able to provide care to patients with hypertension or diabetes?
       – *Explore training, materials, skills, etc.*

HEALTH FAIRS

17. What do you know about the health fairs carried out in this community/region?
       – *Explore/Ask: How do they work? How often are they done? Who attends health fairs? What type of health conditions do participants in health fairs have? How do they participate?*

18. What do you think is good about health fairs?
       – *Explore issues of organization, personnel, time, attendance, costs*

19. Apart from the complications due to COVID-19 and having to bring many people together in one place, what is bad about health fairs?
       – *Explore issues of organization, personnel, time, attendance, costs*

20. In the event that health fairs can be carried out with all the safety measures due to COVID-19, what else do you think could be done to improve health fairs?
    - *Explore about activities, content, advertising, staff, materials or medication*

21. Aside from health campaigns, what do you think would be the best ways to reach people in this community with health information or medical screenings?
    - ➔ **Explore** other specific strategies for hypertension and/or diabetes
