# Supplementary material for: Health system barriers to hypertension care in Peru: Rapid assessment to inform organizational-level change
Source: PLOS Glob Public Health. 2024 Aug 19;4(8):e0002404. doi: 10.1371/journal.pgph.0002404 (PMC11332938; doi:10.1371/journal.pgph.0002404)
Supplement: Attachment — Submitted filename: Response to Reviewers- ANDES Rapid Assessment.docx [file pgph.0002404.s006.docx]

PGPH-D-23-01538
Health system barriers to hypertension care in Peru: Rapid assessment to inform organizational-level change
PLOS Global Public Health

**Responses to Reviewers**

Reviewer #1:

Abstract and introduction

1. Line 76-79: Reference

We have added the following reference:

Byiringiro S, Ogungbe O, Commodore-Mensah Y, Adeleye K, Sarfo FS, Himmelfarb CR. Health systems interventions for hypertension management and associated outcomes in Sub-Saharan Africa: A systematic review. 2023 Jun 8;3(6):e0001794.

2. Line 68- 85: It would be ideal if the flow of the content follows literature review globally, within LMIC, Latin America and then Peru

Thank you for this suggestion. We have reorganized the introduction as suggested.

3. Line 134-136: Could you please provide a rationale for provision of information for anaemia in children (<12 months old) for an article that focuses on hypertension

Our intention was to provide background information about the existing community health worker situation in Peru as context on the health system. We have revised the sentence to more clearly indicate that existing CHWs in the country only focus on anemia and are not trained to address hypertension or other chronic diseases.

“Most existing CHWs focus primarily on controlling anemia in children <12 months old as part of the governmental *Meta 4* program. CHWs in Peru are not currently trained to address hypertension or other chronic diseases.”

Methods

4. Line- 166-167: Could you please provide a reference for the draft theories of change that the research team presented?

We have included the draft theory of change in the supplemental materials and added a reference to it in this sentence.

5. Line 182-188: Could you please add a table for sampling strategy of all participants including no of each participant, type of interview, selection of participants etc.

We have added tables showing characteristics of interview and workshop participants to the supplemental materials. We added detail on the process of selection to the methods section of the manuscript (see below). We did not include participant selection in the table given that all participants for each research activity were selected through the same process.

For workshop participants:

“Participants were recruited by a contact in DIRESA who purposefully selected professionals who had leadership responsibilities in the area of hypertension care, represented various provider types, and came from different regions of Puno.”

For interview participants:

“Participants were purposefully recruited from healthcare facilities in rural and urban areas of Puno that ranked highest in number of hypertensive cases reported according to DIRESA data, including both hospitals and lower-level healthcare establishments. We recruited healthcare professionals in those facilities who provided care for hypertensive patients, seeking variation in the characteristics theorized to be associated with outcomes of interest [21], including range of experience (number of years working at the facility), positions of leadership, and type of provider.”

Table S1. Characteristics of workshop participants.

|  | **Total** | **# Rural** | **# Urban** | **# hospitals** | **# health clinics** | **# MINSA or DIRESA** |
| --- | --- | --- | --- | --- | --- | --- |
| Nurses | 15 | 6 | 9 | 3 | 11 | 1 |
| Doctors | 10 | 3 | 7 | 1 | 5 | 4 |
| Dentists | 6 | 3 | 3 | 0 | 6 | 0 |
| Nutritionists | 1 | 0 | 1 | 0 | 1 | 0 |
| Biologists | 1 | 1 | 0 | 0 | 1 | 0 |
| Midwives | 1 | 0 | 1 | 0 | 0 | 1 |
| Total | 34 | 13 | 21 | 4 | 24 | 6 |

* Provinces covered included Carabaya, Yunguyo, Sandia, Collao, Melgar, Azángaro, Lampa, Chucuito, Puno, San Roman, Huancané

Table S2. Characteristics of in-depth interview participants.

|  | **Total** | **# Rural** | **# Urban** | **1 year or less at facility** | **>1 year at facility** | **Leadership position** | **Non-leadership** |
| --- | --- | --- | --- | --- | --- | --- | --- |
| Nurses | 7 | 6 | 1 | 2 | 5 | 1 | 6 |
| Doctors | 12 | 9 | 3 | 9 | 3 | 2 | 10 |
| Midwives | 1 | 1 | 0 | 0 | 1 | 1 | 0 |
| Dentists | 1 | 1 | 0 | 0 | 1 | 1 | 0 |
| Total | 21 | 17 | 4 | 11 | 10 | 5 | 16 |

6. Line 201- 203: Was the inter-coder reliability checked in Atlas Ti.? Or were there any other methods used to check the reliability? How was the validity measured?

All interviews were coded by the same coder (AFH) and all workshop transcripts were coded by the same coder (KNW). All coded text was reviewed by a small group of authors (KNW, JTM, KCB, AFH, JG) who met regularly to discuss the coding. When there was disagreement over a code assigned to a quote, the group discussed and came to consensus together over which code or codes were most applicable. There were no instances in which a consensus could not be reached.

In this qualitative study, we sought to establish credibility and internal generalizability through consistency in data collection (following a clear protocol for research procedures), triangulation (using multiple methods to cross-verify findings and engaging participants with varying roles and locations), and member checking (through which key workshop and interview participants provided feedback and confirmed the findings).

We have added these descriptions to the Methods section of the manuscript, in the “Data analysis” sub-section.

Results, discussion, conclusions

7. Could you please give some policy-specific examples of how the study informs the current policies that addresses hypertension. (For. E.g., Peru’s National Health Strategy for Control and Prevention of Non-Communicable Diseases, 2004)

We have added a policy implications section to the manuscript and included details around how our findings can inform modifications to the following national guidelines:

(IETSI) IDEDTESEI. GUÍA DE PRÁCTICA CLÍNICA PARA EL MANEJO DE LA HIPERTENSIÓN ARTERIAL ESENCIAL. 2022.

Ministerio de Salud (MINSA). Guía Técnica: Guía de Práctica Clínica para el Diagnóstico, Tratamiento y Control de la Enfermedad Hipertensiva. 2015.

8. Is there any role of private health care system? If yes, could you please explain

We have added to the methods section that only 0.4% of the Puno population is covered by private healthcare insurance. Given that the private system thus would only impact a very small percentage of the population, we have not included discussion about the private healthcare system in our paper. In support of this perspective, the private healthcare system was not mentioned by participants in our workshop or interviews.

9. Could you please provide limitations of the study?

We have added the sub-heading “Strengths and Limitations” to the discussion section to more clearly indicate the limitations that we had previously presented. We have also added some additional limitations. The limitations now read as follows:

“However, our study also has some limitations. Although we included a range of provider types from different types of facilities and areas of Puno, all participants were healthcare professionals. Perspectives of patients, community members, and community health workers must also be considered to obtain a more complete understanding of both supply- and demand-side facets of the healthcare system. Additionally, while the policy implications we developed are based in part on participant recommendations, our paper does not explicitly identify participant-developed solutions to the reported barriers. Furthermore, the workshop and interviews were conducted in late 2021 to early 2022 when healthcare systems were still focusing on COVID-19 and operating under pandemic policies. It is possible that operating procedures and priorities could change as the country progresses post-pandemic.”

Data and supporting information

10. Line 157-159: Could you please add PAHO’s (WHO’s) Rapid health system review template to the annexures for reference? Were there any modifications to the template based on the Peru’s context?

This document is available online at: <https://iris.paho.org/handle/10665.2/50887> (Anexo 2, page 23). An English version of the document is also available here: <https://iris.who.int/handle/10665/275728> (Annex 2, page 21). We previously cited the Spanish version of the document and the link to the document is included in that citation. We have now added a citation with a link to the English version as well into our paper. No modifications were made to the template.

11. Line 189-190: Could you please add the semi-structured interview guide to annexures

We have added this to supplementary materials.

12. Could you please provide an English version of Figure 1. Example theory of change with group comments

We have added an English translation of the figure to supplemental materials.

Reviewer #2: I am grateful for the invitation to review the manuscript "Health system barriers to hypertension care in Peru: Rapid assessment to inform organizational-level change".

The study combines qualitative methodologies to offer a comprehensive and detailed look at the main barriers to hypertension care in a specific area of Peru at the organizational level. The study is clearly justified, uses an appropriate methodology to meet its objectives and is clearly written.

Therefore, most of my comments are suggestions to improve the text and its clarity.

METHODS

1. In the description of the "study setting" include information on poverty in Puno and how it ranks nationally.

We have added the following information: According to the World Bank, Puno had a poverty rate of 8.3% in 2023, ranking highest out of all regions of Peru.

**Citation:** World Bank. Geospatial Poverty Portal. World Bank Group. 2023. www.pipmaps.worldbank.org (accessed January 2023).

2. In the description of the "Peruvian health care system" mention health insurance and its coverage in Peru. It would also be key to understand which services related to hypertension are covered by the insurance (i.e. care, medications, tests, etc.) and which are usually out-of-pocket expenses.

We have added the following information about health insurance coverage in Puno to the site description section: “Most people in Puno (65%) receive health insurance through the national governmental insurance scheme (Seguro Integral de Salud, or SIS); 12% are covered by EsSalud, 0.7% by the armed forces or police, 0.4% through private insurance, 0.4% through another scheme, and 35% are uninsured.”

We have also added details on what is covered by insurance to the site description section:

“Insurance covers all medical care and medication costs, but in the event of stock-outs, patients must pay out-of-pocket for medications at private pharmacies.”

3. Any thoughts on the role of traditional medicine in the region?

We have added the following to the site description: “Traditional healers using medicinal plants and spiritual treatments for various conditions, including heart problems and circulatory system disorders, are also common in the Andean region of Peru. However, recent research suggests high acceptance of modern medicine.”

**Citations:**

Bussmann RW. The globalization of traditional medicine in northern Peru: From shamanism to molecules. 2013:291903. <https://doi.org/10.1155/2013/291903>.

Williamson J, Ramirez R, Wingfield T. Health, healthcare access, and use of traditional versus modern medicine in remote Peruvian Amazon communities: A descriptive study of knowledge, attitudes, and practices. Am J Trop Med Hyg. 2015;92(4):857-864. doi: 10.4269/ajtmh.14-0536.

4. This sentence from the description of the health system requires a reference: “After diagnosis, routine hypertension follow-up care can be provided at lower-level facilities, although some patients prefer to seek routine care from a cardiologist”.

This statement was based on personal experience by the Peruvian doctors on our authorship team. We have deleted the statement given that it is anecdotal.

5. If available, include the number of cardiologists in the Puno region, so that the reader can understand the situation of the health system and the scarce supply of specialists in Puno.

We could not find data on the number of cardiologists in Puno. We have deleted the specific reference to cardiologists given the anecdotal nature of the information. However, we do have information on the provider-to-patient ratio in the site description:

“…rural areas of Puno still face severe human resource shortages (1 physician per 689 people in 2020) and challenges with high staff turnover.”

Instituto Nacional de Estadística e Informática, (INEI). Recursos humanos de salud. https://m.inei.gob.pe/estadisticas/indice-tematico/health-human-resources/. Updated 2022.

6. In the characterization of the participants in the workshop, add information about their roles (in addition to their profession). In the case of the interviews, you do detail if they have "leadership positions".

We have added tables with participant information to supplementary materials.

7. In "Data Analysis" describe the procedure for the generation of the (sub)codes within the 6 themes.

We have added the following sentence to describe identification of sub-themes: “The small author group identified emerging sub-themes by grouping similar quotes together within each theme.”

RESULTS

8. A general comment is that the results section, which is about 10 pages long, feels a bit long and could be shortened. It's not essential, so I leave that up to the editor.

We have cut some text throughout the results section to reduce the length.

9. Although the barriers identified are at the organizational level, I wondered if some were associated with gender (e.g., service hours may be a more problematic issue for men than for women?) and if there were any perceived differences between the two majority ethnic groups in Puno: the Aymara and the Quechua, regarding the barriers. If identified, it would be relevant to include them in the results.

This is an interesting point. The issue of gender differentially influencing accessibility to healthcare did not arise in our interviews or workshop with healthcare providers. Providers also did not distinguish between Aymara and Quechua when discussing cultural challenges. These are interesting topics that our team will seek to explore further in future research.

10. In the table describing the barriers it does not make sense to include the following: "Integration of CHWs into the healthcare system could improve care". That seems to be more of an opportunity and I understand that it is more aligned -and perhaps also biased? - to questions the authors asked to design their intervention.

We have removed that point from the table.

11. In the results section, when talking about social programs, it is not accurate to say that JUNTOS is not integrated with health goals. As far as I remember, it is a conditional transfer program, and one “condition” is the children's health. In any case, the sentence should be more precise.

We have revised the sentence to better reflect that this was an opinion reported by the participants: “While financial assistance is given through government social programs such as Pension 65 (for those 65 and older) and Juntos (for poor families with young children), some participants believed that the money is sometimes used to purchase unhealthy, high caloric content foods.”

DISCUSSION

12. Are there some barriers that do not appear in your study and do appear in other studies in similar / different settings? Are there barriers that were expected and did not appear in your study? Could the informants chosen have something to do with these absences (i.e. not having service users)?

The reviewer correctly notes that we did not include service users (i.e., patients) in our study, and thus, the barriers we identified were mainly systems-level issues recognized by providers. Previous research that has included patient-generated barriers to hypertension control have found factors such as affordability of medical care and medications, motivation and capacity to adopt healthy lifestyle changes, knowledge about the importance of controlling hypertension, and perceived acceptability of healthcare services are key barriers to hypertension management (Khatib et al., 2014). These factors did not arise in our study given that we were focused on the health system and only included healthcare providers.

We have added these details to the discussion section.

Khatib R, Schwalm JD, Yusuf S, et al. Patient and healthcare provider barriers to hypertension awareness, treatment and follow up: a systematic review and meta-analysis of qualitative and quantitative studies. PLoS One. 2014;9(1):e84238. Published 2014 Jan 15. doi:10.1371/journal.pone.0084238

13. In the introduction section, one of the objectives of the qualitative work is detailed as "to inform the design of an intervention to be tested in a randomized controlled trial". It would be interesting for one of the paragraphs of the discussion to reflect on the decisions that were derived from these results to design the intervention. This would allow us to reflect on the value of these formative studies.

The rapid assessment and health provider interviews presented in this paper are just one of several formative research activities that were undertaken for the ANDES trial. Intervention design decisions were made based on findings from all of these research activities. Thus, we feel it is beyond the scope of this paper to present decisions which were based on a larger body of formative evidence. Our protocol paper, which was recently accepted to the journal *Implementation Science Communications*, summarizes our intervention plan based on the full set of formative findings. We will add a citation to that protocol paper as soon as it is available.

14. The discussion states that "interventions that shift some responsibilities to health facility providers, such as CHWs, could be an effective way to improve healthcare in the area". But the results themselves indicate that their inclusion presents challenges. The discussion should reflect on mechanisms to overcome these challenges - not only their training but their inclusion within the system.

This is a good point. We have added the following sentence into the discussion:

“However, to be effective, CHWs must be integrated into and recognized as a formal part of the health system, including adequate renumeration, training, supervision, and inclusion within national policies and guidelines such as the Practical Clinical Guide for the Management of Hypertension and the Technical Guide for the Diagnosis, Treatment, and Control of Hypertension.”

15. Two important absences -or that would have been an important contribution- are that the participants proposed measures to address the barriers, and having the viewpoint of decision-makers and users.

Thank you for noting this. We agree. We had previously noted in our limitations section that this paper does not include perspectives of patients, community members, or community health workers. We have revised to more clearly flag this as a limitation. We also added a sentence noting that a limitation of our paper is that we did not include participant-developed solutions to the identified barriers. The section now reads:

“Although we included a range of provider types from different types of facilities and areas of Puno, all participants were healthcare professionals. Perspectives of patients, community members, and community health workers must also be considered to obtain a more complete understanding of both supply- and demand-side facets of the healthcare system. Additionally, while the policy implications we developed are based in part on participant recommendations, our paper does not explicitly identify participant-developed solutions to the reported barriers. Furthermore, the workshop and interviews were conducted in late 2021 to early 2022 when healthcare systems were still focusing on COVID-19 and operating under pandemic policies. It is possible that operating procedures and priorities could change as the country progresses post-pandemic.”

16. There are two ideas from the findings that I believe should be part of the discussion, in a subsection on policy implications: "For example, policy makers could revise national guidelines on hypertension to take into account local cultural beliefs and resource availability, and integrate health considerations into all social programs. Drug distribution systems could be redesigned to avoid problems of overstocking and understocking, which could be helped by more cohesive and integrated electronic health information systems."

Thank you for this useful suggestion. We have added a section to the discussion on policy implications and integrated these points.
