# Supplementary material for: Health system barriers to hypertension care in Peru: Rapid assessment to inform organizational-level change
Source: PLOS Glob Public Health. 2024 Aug 19;4(8):e0002404. doi: 10.1371/journal.pgph.0002404 (PMC11332938; doi:10.1371/journal.pgph.0002404)
Supplement: Attachment — Submitted filename: ANDES Rapid Assessment Response to Reviewers 2.docx [file pgph.0002404.s007.docx]

PGPH-D-23-01538
Health system barriers to hypertension care in Peru: Rapid assessment to inform organizational-level change
PLOS Global Public Health

**Responses to Reviewers #2**

They recommend improving the results section, specifically by discussing potential additional barriers, and discussing potential evidence for other unhealthy behaviours. They additionally recommend extending the discussion section, by discussing how the results fit in with the existing body of knowledge.

Thank you for the suggestions. We have reformatted the results section to more clearly show where we discuss each of the key barriers from Table 1. We have also clarified that our use of the term “healthy lifestyles” was intended to refer to both healthy diets and exercise. Furthermore, we have expanded the section on financing to indicate not only the lack of funding for hypertension diagnosis and management, but also for hypertension awareness and prevention. We have also expanded our discussion section to explain why greater investments in hypertension awareness raising/prevention and improved management is essential, including new citations to literature demonstrating these benefits. We additionally added a paragraph to the discussion section highlighting how our results fit within the existing body of literature. Please see our responses to the specific reviewer comments below for details on these changes.

Journal Requirements:

1. Please review your reference list to ensure that it is complete and correct. If you have cited papers that have been retracted, please include the rationale for doing so in the manuscript text, or remove these references and replace them with relevant current references. Any changes to the reference list should be mentioned in the rebuttal letter that accompanies your revised manuscript. If you need to cite a retracted article, indicate the article’s retracted status in the References list and also include a citation and full reference for the retraction notice.

Please see the list below of the new references we have added to the manuscript.

[24] Samb B, Desai N, Nishtar S, et al. Prevention and management of chronic disease: A litmus test for health-systems strengthening in low-income and middle-income countries. *The Lancet*. 2010;376(9754):1785-1797. doi: 10.1016/S0140-6736(10)61353-0.

[25] Topp SM, Abimbola S, Joshi R, Negin J. How to assess and prepare health systems in low- and middle-income countries for integration of services—a systematic review*. Health Policy Plan*. 2018;33(2):298-312. doi: 10.1093/heapol/czx169.

[26] World Health Organization. Fact sheet: Noncommunicable diseases. https://www.who.int/news-room/fact-sheets/detail/noncommunicable-diseases. Updated 2023.

[30] Badanta B, Lucchetti G, Barrientos-Trigo S, et al. Healthcare and health problems from the perspective of indigenous population of the Peruvian amazon: A qualitative study. *International Journal of Environmental Research and Public Health*. 2020;17(21). doi: 10.3390/ijerph17217728.

[31] Díaz-Ruiz R, Vargas-Fernández R, Rojas-Roque C, Hernández-Vásquez A. Socioeconomic inequalities in the use of medical consultation services in Peru, 2019. *International Journal for Equity in Health*. 2024;23(1):10. doi: 10.1186/s12939-024-02099-2.

[32] Kostova D, Spencer G, Andrew EM, et al. The cost-effectiveness of hypertension management in low-income and middle-income countries: A review. *BMJ Global Health*. 2020;5(9):e002213. doi: 10.1136/bmjgh-2019-002213.

2. We have noticed that you have uploaded Supporting Information files, but you have not included a list of legends. Please add a full list of legends for your Supporting Information files after the references list.

We have added a list of the Supporting Information after the References section.

Reviewer #1: Results:

• Not all key themes identified in Table 1 are addressed in the results section, potentially causing difficulty in following the results section. It may be helpful to specifically number and elaborate on each theme.

Thank you for this recommendation. We have added numbers into the table and in the results section to more clearly highlight where each of the themes from the table is covered in the text.

• The evidence presented under "National guidelines are incompatible with the local context" suggests only financial constraints, rather than cultural or language barriers, are the primary issue.

The quote we included from the in-depth interviews for this section specifically elucidates the point about how resource availability (i.e. financial constraints) makes it difficult for patients to follow national guidelines. Discussions at the workshop also mentioned cultural and language barriers with applying national guidelines in Puno. However, we do not have quotes from the workshop given that the workshop was not recorded. Figure 1 (and the English version in Figure S2) highlight some of the cultural and language barriers that were mentioned at the workshop and how national clinical guidelines need to be adapted based on the reality in Puno. We added a note that examples of those barriers can be seen in Figure 1 (Spanish version) or Figure S2 (English version).

• While the study focuses on promoting a healthy diet for managing hypertension, it's unclear if there is evidence addressing other unhealthy behaviors such as smoking, alcohol consumption, physical inactivity.

Our participants focused mainly on healthy diets and exercise for preventing hypertension. We have clarified in the section “Lack of national-level integration of hypertension prevention into policies” that by healthy lifestyles, participants mentioned diet and exercise.

“Participants stated that there is little value placed on the promotion of healthy behaviors at a societal level and there has been little effort to make rural environments more conducive to healthy lifestyles (including healthy diets and exercise).”

“Multiple providers stated that raising awareness of healthy eating, healthy lifestyles (i.e., exercise), and awareness about hypertension is needed”.

• Regarding "Limited funding for hypertension care," does the study provide evidence of funding allocation specifically for hypertension awareness, considering participants' suggestions for improved database and tracking? Clarifying the significance of raising awareness and its potential impact could enhance the study's depth.

Thank you for recognizing this. We have updated the section to specifically note that participants mentioned very little funding is available for hypertension in general, including for awareness and prevention.

“This leaves little funding for diagnosis and management of hypertension, as well as for hypertension awareness and prevention.”

We have also added the following sentence to our discussion section to highlight the importance of raising awareness of hypertension:

“Our findings also suggest the need for greater investment both in raising awareness of hypertension and healthy lifestyles for prevention and early diagnosis (as lack of knowledge is a commonly reported barrier to hypertension detection and control [7]), as well as for improving management and treatment of the disease (as controlling hypertension has been shown to be highly cost-effective [27]).”

• What are the three different levels of the health system, and is the data used for decision-making at each level?

Participants mentioned three different health information systems that are used within the same health facilities. We have added information into the paper on what the systems are and who they are managed by. However, participants did not elaborate on how the data from these different systems were used or if/how they are used for decision-making.

We revised the section accordingly:

“According to participants, there are three different and incompatible health care data management systems used in health facilities (the HIS system managed by the national Ministry of Health, the SIS system managed by the national health insurance program, and a registry system managed by the national vital records body). Barriers noted from the use of these different systems within the same health facility included inconsistent data collection and challenges with standardization and integration.”

Discussion:

• The discussion section, being only one page, may be considered relatively short. The decision is left to the editor's discretion.

We have added two paragraphs to the discussion section highlighting how our results fit within the existing body of literature and alignment with broader healthcare disparities in Peru. Previous reviewers had asked that we structure our discussion section to specifically highlight the policy implications in a sub-section of the Discussion. We consider this to be an important part of the discussion given that we are discussing the broader relevance of our findings.

• How do the findings of this study contribute innovatively to the existing body of knowledge on health system assessments?

Thank you for this suggestion. We have added a paragraph to the discussion section to highlight the importance and innovativeness of our study.

“The global agenda for health systems strengthening has historically focused mainly on specific infectious diseases such as HIV, tuberculosis, malaria, and vaccine-preventable diseases [24]. In line with this, most existing health systems assessments conducted in LMICs have focused on the ability of primary healthcare settings to address and integrate care for communicable diseases [25]. Also, research on responses to chronic disease often focus on tertiary, curative, and specialist care [24]. Given that noncommunicable diseases, including hypertension, are responsible for 41 million deaths globally each year, 77% of which are in LMICs, there is an urgent need to identify strategies for improving the healthcare system in LMIC settings to address this issue [26]. Our study innovatively explores how chronic disease (namely, hypertension) is considered within the Peruvian primary healthcare system, highlighting how health systems could be strengthened to better address chronic disease.”

• Are the identified barriers to hypertension care in Puno inconsistent with the existing literature on healthcare systems in similar settings?

We have added a sentence to our discussion section to highlight that the challenges we found that primary health facilities face in dealing with chronic disease are similar to health system challenges for communicable diseases.

“This study demonstrates that challenges faced by the health system in dealing with chronic disease are similar to those faced for communicable diseases. For example, medication shortages and distribution challenges are common barriers to treatment adherence in LMICs [6]. Other studies have similarly found that a lack of culturally competent care and increased distance from healthcare facilities deter healthcare seeking behavior [28], and lack of coordination across different healthcare providers (i.e. due to inadequate clinical information systems or lack of integrated platforms) are common challenges to continuity of care [29].”

• How do the identified barriers align with the broader context of healthcare disparities and challenges in Peru?

Thank you for this suggestion. We have added a paragraph to our discussion section to describe how our findings relate to broader healthcare disparities in Peru.

“Our findings underscore important healthcare disparities in Peru. First, rural populations of Peru experience greater limitations in access to healthcare compared to urban populations [13]. Our research, conducted in the rural area of Puno, highlighted such challenges with rural healthcare infrastructure, including challenges with the supply and distribution of medications, lack of equipment, and shortages of healthcare providers. Second, indigenous populations often face ethnic and cultural disparities in access to healthcare [27], as our study similarly found. Third, lower-income populations face socioeconomic barriers to accessing healthcare [28]. Our study underscores the further barriers that the low-income population of Puno face when attempting to access hypertension care. For example, lower-income populations are more affected by medication shortages given that they are unable to purchase medications from private pharmacies.”
