# Supplementary material for: Health system barriers to hypertension care in Peru: Rapid assessment to inform organizational-level change
Source: PLOS Glob Public Health. 2024 Aug 19;4(8):e0002404. doi: 10.1371/journal.pgph.0002404 (PMC11332938; doi:10.1371/journal.pgph.0002404)
Supplement: Attachment — Submitted filename: ANDES Rapid Assessment Response to Reviewers 3.docx [file pgph.0002404.s008.docx]

PGPH-D-23-01538
Health system barriers to hypertension care in Peru: Rapid assessment to inform organizational-level change
PLOS Global Public Health

NOTE: New references in the reference list are highlighted in yellow.

**Responses to Reviewers #3**

- Line 73: clarify these %s are out of those living with hypertension, not all adults

Thank you for clarifying this. We have changed the sentence to: “In Latin America and the Caribbean, only an estimated 35% of women and 23% of men with hypertension have their blood pressure under control [4].”

- Line 114: add date of the data cited (and are these the most recent statistics prior to time of the study?). SIS eligibility has expanded (to the point that MINSA considers the full population to be covered, even while individuals may report lack of insurance), so it is important to contextualize this evidence.

We have updated the data to 2022 statistics, which are the most recent data available through the national statistics body of Peru (Instituto Nacional de Estadística e Informática- INEI). The sentence now reads: “As of 2022, most people in Puno (66.6%) receive health insurance through the national governmental insurance (Seguro Integral de Salud, or SIS); 11.2% are covered by EsSalud, 0.6% through other insurance (i.e., armed forces, police, or private insurance), and 21.6% are uninsured [11].”

- Line 129 - It may be more appropriate to say 'Blood pressure should be measured' in defining the guidelines, given gaps between guidelines and practice

Thank you for this suggestion. We have changed the sentence to “Blood pressure should be measured” as suggested.

- Lines 135 - 143: Reviewer 1 raised a concern around the inclusion of anemia here; I agree that the information on CHW roles (or lack of role) for hypertension is not a methodological component of the paper to the extent that it is presented here, and should be described more briefly, with elaboration in the Discussion section as desired.

We have deleted the following sentences:

“Most existing CHWs focus primarily on controlling anemia in children <12 months old as part of the governmental Meta 4 program. CHWs in Peru are not currently trained to address hypertension or other chronic non-communicable diseases.”

We edited an earlier sentence to more briefly mention that CHWs in Peru do not currently focus on chronic disease: “…however, their training and activities are usually focused on specific health topics, and they are not currently trained to address chronic disease.”

- Line 222: "internal generalizability" - suggest "internal validity" as a more clear and familiar term

We have changed this to “confirmability”, which is a more widely used criteria for evaluating the trustworthiness of qualitative research.

Tobin, G.A. and Begley, C.M. (2004), Methodological rigour within a qualitative framework. Journal of Advanced Nursing, 48: 388-396. https://doi.org/10.1111/j.1365-2648.2004.03207.x

The revisions to the discussion section further strengthen this element of the manuscript; however, they rely primarily on review or overview articles across LMICs, with more limited engagement with what is already known and attempted for CVD control in Andean Peru. I would suggest further engagement with country or population-specific literature to enrich this section. Articles that touch on similar barriers to those identified in this manuscript include:
<https://link.springer.com/article/10.1007/s11906-023-01286-w>
<https://www.tandfonline.com/doi/full/10.1080/16549716.2021.1975920>
<https://www.ssph-journal.org/journals/international-journal-of-public-health/articles/10.3389/ijph.2021.1604117/full>

Thank you for this suggestion and the article links. We have added references to the suggested manuscripts to our discussion section, as pasted below:

“Another study in Peru also found that primary health care facilities were underfunded (especially for non-communicable diseases), and that surveillance systems were unable to accurately track the disease burden to justify higher budgets [29].”

“Other work in Peru has also found that clinical guidelines were not adapted to the local context, healthcare workers lacked training on chronic diseases, and shortages of medication and appropriately calibrated devices were lacking across primary care facilities [29].”

“In addition to raising awareness, previous research in Peru has demonstrated the importance of removing barriers to healthy lifestyles (i.e. limited availability and high cost of healthy food, gendered barriers to engaging in sports, geographic distance to healthcare centers, and poor treatment by doctors) [35].”

“These recommendations could be integrated into Peru’s efforts to scale up the WHO HEARTS initiative to improve hypertension care in primary healthcare settings [37].”
